# Religion and the everyday citizenship of people with dementia in Nigeria: A qualitative study

**DOI:** 10.4102/ajod.v13i0.1338

**Published:** 2024-03-31

**Authors:** Elizabeth O. George, Ruth L. Bartlett

**Affiliations:** 1 Centre of Diaconia and Professional Practice, VID Specialized University, Oslo, Norway; 2 Faculty of Health Studies, VID Specialized University, Oslo, Norway; 3 School of Health Sciences, University of Southampton, Southampton, United Kingdom

**Keywords:** dementia, citizenship, religion, Africa, lived experience

## Abstract

**Background:**

Research on the lived experience of dementia is burgeoning across the social and health sciences. Yet, very little is still known about the experience of dementia for many tribes and ethnoreligious groups, as most studies are conducted in Western countries.

**Objective:**

The aim is to advance the understanding of the role of faith and prayer in the lives of people with dementia in Nigeria through a lens of everyday citizenship.

**Method:**

Interviews were conducted with 17 older people with dementia in a low-income, Yoruba-speaking community in Southwestern Nigeria. After transcription, the data were analysed thematically.

**Results:**

The major theme identified in participants’ accounts was that prayer served as a space for active and agentic participation. This theme was further elaborated upon through four subthemes: (1) agency in routine and daily prayer, (2) cognitive (re)framing through prayer, (3) prayer as a vehicle for active social interaction and support, and (4) prayer as work and transaction.

**Conclusion:**

Participants described religious practices as important to their acceptance of the situations, their feelings of hope in everyday lives, and their connection and contributions to the community. Analysis also shows the centrality of relationality in the everyday experiences of people with dementia.

**Contribution:**

This article contributes to advancing the understanding of the socially orientated everyday experience of dementia. It contributes to a small body of literature on the social aspect and everyday experiences of living with dementia in Africa and stands out as the first of its kind study in Nigeria.

## Introduction

Dementia is one of the leading causes of disability among older people globally (World Health Organization 2021). Dementia is an umbrella term for a collection of symptoms caused by neurological conditions affecting the brain and impacting memory, thinking, behaviour, and emotion. The most common form of dementia is Alzheimer’s disease, affecting 50% – 60% of people with dementia (Alzheimer’s Disease International 2023). Other forms include Lewy body dementia and vascular dementia. The cognitive and physical impairments caused by dementia are long-term and constitute a disability under the United Nations Convention on the Rights of Persons with Disabilities (CRPD) (UN General Assembly 2007). It is estimated that 55 million people live with some form of dementia, with more than 60% in low- and middle-income countries (WHO 2021). Evidence suggests that there are over 2.13 million living with dementia in sub-Saharan Africa, with a projected increase of 257% by 2050 (Guerchet et al. 2017).

Research to date has focused on finding a cure and understanding the biomedical markers and impacts of living with dementia (Guerchet et al. 2017; World Health Organization 2021). Medicalised understandings of dementia have dominated the global scientific community for decades (Fletcher 2023; Kitwood 1997). In Africa, while dementia remains generally mischaracterised and underdiagnosed in the general public (Adebiyi et al. 2016; Brooke & Ojo 2020), within dementia scholarship, dementia is dominantly approached as a ‘disease’ whose cause, prevalence, and clinical management need to be understood and addressed (see, e.g., Amoo et al. 2011; Olakehinde et al. 2019; Yusuf et al. 2018). While this work is important, it positions people with dementia only as ‘patients’ with a health condition, so broader understanding of how people with dementia navigate the experience is lacking.

In recent years, efforts to understand the lived experience of dementia have improved as the perspectives and voices of people with dementia are increasingly sought. However, most of this work has been conducted in the West (notably Canada, the UK, Australia, and Nordic countries); the lived experience of dementia among tribes and ethnoreligious groups is notably absent from this scholarship. Take, for example, the impressive set of meta-syntheses involving a review of 169 articles in total related to the lived experience of social relations (Eriksen et al. 2016), space (Førsund et al. 2018), and time (Eriksen et al. 2021) among people with dementia; not one of the included studies were conducted in an African country, and only three were conducted outside the West (in China, Iran, and Pakistan). Only a handful of researchers have sought the perspectives of people with dementia living in Africa (see, e.g., Agyeman et al. 2019; Hindley et al 2017; M’belesso et al. 2016). To the best of our knowledge, none of these studies have been conducted in Nigeria. This article, thus, fills a gap in knowledge by exploring, from the perspectives of people with dementia, their experience of living with dementia in Nigeria.

In Nigeria, there are several significant limitations to dementia research, care, and policies (Adeloye et al. 2019; Nwakasi et al. 2021). Statistics show that the number of Nigerians with dementia is increasing, with an estimated increase of over 400% between 1995 and 2015 (Adeloye et al. 2019). However, there is no National Strategy or Action Plan for dementia care and support in Nigeria (Ayinde & Ogundele 2019). While Nigeria is a signatory to the CRPD, people with dementia are not included in disability rights activism in Nigeria. In addition, dementia-related scholarship in Nigeria appears to be skewed towards medical approaches (e.g., Amoo et al. 2011; Ochayi & Thacher 2006; Olakehinde et al. 2019). While socially orientated perspectives on dementia in Nigeria are evident, they mainly focus on the views of caregivers and community members (Adebiyi et al. 2016; Nwakasi et al. 2021; Yusuf & Baiyewu 2012), with the perspectives of people with dementia significantly missing.

Studies conducted in Nigeria and Africa have highlighted religion’s role in the (predominantly negative) perception of and attitudes towards people with dementia (Brooke & Ojo 2020; Hindley et al. 2017; Ogunniyi et al. 2005). However, the voices of people with dementia are missing from these studies, as the experience of religion is reported from the perspective of carers and traditional and/or faith leaders. Similarly, when spirituality is explored from the perspective of people with dementia, there is an overrepresentation of Euro-Christian perspectives (e.g. Beuscher & Grando 2009; Dalby, Sperlinger & Boddington 2012; Jolley et al. 2010; Katsuno 2003; Snyder 2003); African Christian, Islamic, and traditional religious perspectives are significantly lacking. The aim of this article is to advance the understanding of the role of faith and prayer in the lives of people with dementia in Nigeria through a lens of everyday citizenship. We define everyday citizenship here as agentic activities that people with dementia partake in, in everyday and ordinary settings, as members of their families and communities. Faith and prayer are rarely connected to the notion of everyday citizenship, even though they are both integral to social participation, especially communal prayers. Thus, this article serves as a much-needed counterbalance to Westernised notions of life with dementia and the spiritual aspect of people’s lives.

### Linking lived religion to everyday citizenship

In the scholarly world, religious beliefs and practices are commonly regarded as existing in a domain completely removed from practical and everyday life (Rubin, Smilde & Junge 2014). Nevertheless, religious beliefs and practices can – and do – have a place in every area of people’s social lives, even when they are generally based on the existence of supernatural or otherworldly powers (Riesebrodt 2010). Consider, for example, how Islamic beliefs influence daily routines in many towns in Northern Nigeria, such as when to wake up, living arrangements, architectural designs of homes, family formation, marriage, social relations with family and non-family members, child rearing, in-school and after-school activities, market opening hours, money lending practices, burials, inheritance, and other aspects of home and public life. In this study, ideas and concepts related to lived religion and everyday citizenship are uniquely intertwined to provide a theoretical compass for understanding people’s experiences of living with dementia in Nigeria.

Lived religion refers to the ways in which people utilise and are influenced by religion in their day-to-day lives (Ammerman 2007; McGuire 2008; Reimer-Kirkham 2009). Religion, as daily lived and practised in private and public domains, often reflects in communities and the society at large, visible in the forms of moral and/or ethical codes and ways of relating with others who are expected to be members of the religious community. For instance, in a study including participants with diverse religious and spiritual representations – such as Christianity, Sikhism, Islam, Hinduism, Judaism, and Atheism – religion was shown to provide a comprehensive code of conduct for life in general, including how to relate with people and what kinds of food to eat and clothes to wear (Reimer-Kirkham 2009). In this sense, ordinary and everyday religious practices become an important site from which to explore and emphasise opportunities for everyday citizenship in the lives of people with dementia.

Everyday citizenship refers to agentic displays and forms of communal support that happen in ordinary (as opposed to political) settings (Nedlund, Bartlett & Clarke 2019). In Nigeria, such displays might range from a person with dementia participating in town hall meetings to address specific communal challenges to choosing whether to continue living in their own homes or relocate to their children’s homes. Ordinary spaces are not usually considered a space for citizenship, but citizenship scholars have begun to draw attention to how interpersonal relations and other structures of rule and belonging can be played out from marginal spaces rather than state agents (e.g. De Koning, Jaffe & Koster 2015; Neveu 2018; Turner 2016). For example, in one Australian study involving 80 Muslims, researchers found that participants enacted everyday citizenship through actively participating in ordinary multicultural spaces (Roose & Harris 2015). In capturing and recognising these routine aspects of social relations and practices, we give importance to them and seriously consider them not only as a category of analysis but also as proof that everyday life experiences and relations are more than just ordinary and unremarkable (Neal & Murji 2015). Thus, by examining the role of ordinary, taken-for-granted religious aspects such as faith and prayer in people’s lives, extraordinary communal dynamics of everyday citizenship may become clearer to see.

## Research methods and design

### Research design

This study aimed to explore the role of religious faith and prayer in maintaining the everyday citizenship of people with dementia in a Nigerian community. It was, therefore, important to adopt a qualitative research design to facilitate an in-depth exploration of the participants’ life world or lived experiences (Creswell & Poth 2016). Qualitative research is particularly well-suited for providing detailed and comprehensive descriptions of human experiences, necessitating researchers to acknowledge their roles as co-constructors of knowledge, which may be influenced by their identities and positionality (Denzin & Lincoln 2011; Sadiki, Watermeyer & Abrahams 2021). Semi-structured interviews were conducted to elicit information from participants. According to Silverman (2013), utilising interviews as part of the qualitative research design is valuable for an in-depth understanding of the phenomenon being investigated within its specific contextual circumstances.

### Study population and data collection

Inclusion criteria included adult persons living in Agbado (not real name), a community in Southwestern Nigeria, with dementia. Using purposive sampling, the community was chosen, and participants were recruited. Access to the community and persons with dementia was facilitated by a Study of Ageing team from the Department of Psychiatry at the University College Hospital in Ibadan, which had ongoing longitudinal research projects in different communities in Ibadan (see, for example, Gureje et al. 2011). The team at the University College Hospital, which had an ongoing dementia assessment project in the community, at the time of data collection, had data on the number of people with dementia in the community, the kinds of dementia they had, and the severity of their dementia diagnosis. As this study was not concerned with details of the medical diagnosis but with the everyday experiences of living with dementia, details of participants’ dementia diagnoses were not collected. The first author, hereafter referred to as the researcher, only asked for access to their patients who had been assessed as having mild to moderate level dementia whom the researcher could communicate with. While the researcher did not conduct the assessment of participants’ dementia themselves, they trusted that participants had dementia as this assessment had been performed by a team of medical professionals who were trained to do that, and participants’ family members knew that they lived with dementia.

Using a household contact information list provided by the team at the University College Hospital, the researcher and a local interpreter with almost 30 years of experience working with the team as a community research assistant visited households with a person with dementia. The contact information list was provided in bits – containing five to seven households at a time, so recruitment and data collection were conducted continuously until saturation was accomplished, that is until no new themes or ideas were arising from the interviews (Saunders et al. 2018). In total, 17 people with dementia were recruited to participate in the study. Before commencing the study, both people with dementia and member or members of their households were presented with full information about the study in Yoruba, Nigerian Pidgin, or English – depending on which language the person preferred to communicate in. Table 1 shows the demographic characteristics of the 17 participants with dementia. The table does not show details of participants’ dementia diagnoses, as this information was not collected. The ages of many participants are only an estimate as neither they nor their families could tell their exact ages.

**TABLE 1:** Demographic characteristics of participants.

S/N	Pseudonyms	Gender	Religion	Age (estimates)	Living arrangement	Other impairment or condition
1	Baba Ade	Male	Muslim	90	Alone but grandchild and great-grandchildren live nearby	Arthritis
2	Mama Bosedee	Female	Muslim	92	With son	Limited mobility
3	Mama Doyin	Female	Muslim	96	With daughter-in-law and grandchild	Paraplegic
4	Mama Enitan	Female	Muslim	90	With co-wife	-
5	Mama Feyi	Female	Muslim	94	With great-grandson	Limited mobility
6	Baba Gbadebo	Male	Muslim, Ìṣẹ̀ṣẹ (Indigenous religion)	85	With wife and children	Limited mobility
7	Mama Ibukun	Female	Christian	96	With daughter	Stroke, paralysis
8	Mama Jaiye	Female	Muslim	94	With son	-
9	Mama Kike	Female	Muslim	85	Alone, with neighbours	-
10	Mama Lola	Female	Muslim	86	With daughter and disabled son	Stroke, paralysis
11	Mama Mayowa	Female	Muslim	93	With son	Visual and hearing impaired
12	Mama Niyi	Female	Muslim	85	With son	Visual and hearing impaired
13	Mama Odunayo	Female	Muslim	80	Alone, with neighbours	-
14	Mama Ronke	Female	Christian	87	With son	Limited mobility
15	Baba Seriki	Male	Muslim	90	With wife	Visually impaired
16	Mama Tani	Female	Muslim	90	With daughter	Limited mobility
17	Mama Yejide	Female	Muslim	90	With husband and children	Limited mobility

The interviews were conducted between February 2022 and May 2022 in a small community in Southwestern Nigeria. Interviews were primarily conducted in Yoruba by the researcher with the aid of an interpreter, in participants’ homes. Interviews lasted approximately 60 min. Some participants were interviewed twice to collect additional information or clarify previously provided information. The interviews were recorded and field notes were written to capture extra information.

### Data analysis

The audio recordings were transcribed by a Yoruba-speaking Nigerian who was instructed to transcribe everything said during each interview in English. Transcribing what participants said and what the interpreter translated helped to ensure that participants were credited with only what they said in the interviews. The transcribed data were subjected to a thematic analysis, which entailed identifying, interpreting, and presenting patterns of meaning within the data (Braun & Clarke 2006; eds. Ritchie et al. 2014). Using an abductive approach – going back and forth between data and theory (Earl Rinehart 2021) – the coding process involved reading the transcripts in conversation with the literature on dementia and citizenship. The transcripts were coded and categorised manually, on paper and using Microsoft Word.

To protect the anonymity of participants, pseudonyms are used. To show the gender of each participant, the honorary title of ‘Mama’ or ‘Baba’ was attached to their pseudonym, which stays true to Yoruba culture, where it is considered rude to address an older person by their name alone. In reporting the quotes, labels are also assigned (Willis et al. 2016) based on participants’ gender and religion. For example, the label ‘Mama Feyi, M’ refers to a female Muslim participant named Feyi.

### Ethical considerations

The study was assessed by the Norwegian Centre for Research Data (SIKT) (Ref number: 227353) following an evaluation by the Regional Committees for Medical and Health Research (REK) (Ref number: 293298). Ethical approval was also granted by the University of Ibadan/University College Hospital Ethics Committee (number: UI/UC/21/0674) in accordance with the National Code for Health Research Ethics in Nigeria. Full and informed consent was a requirement for participation based on the ethical standards listed by the Ethics Boards. Thus, participants were provided with clear and complete information about the study before obtaining their consent. Information about the study was provided in English, Pidgin, and Yoruba, depending on which language participants were more comfortable with. ‘Dementia’ could not rightly translate into Yoruba or Pidgin; phrases such as ‘memory problems or loss mostly associated with ageing’ were used instead.

To ensure that the person with dementia understood what they were consenting to, they were asked questions about what the project was about and what was required of them. Participants with dementia demonstrated an understanding that the researcher had come to speak to them about living with dementia (or as translated to Yoruba: memory problems mostly associated with ageing), and they showed that they understood what it meant to consent to participation. However, because this work was being performed within a culture where decisions are usually approached collectively, it seemed respectful to approach consent and recruitment collectively. Thus, in each household, both the person with dementia and a member of their household – usually a family member or close neighbour – provided verbal consent and signed or gave their thumbprints on the written consent forms.

### Results

The main theme identified in this study was: prayer as a space for active and agentic participation. This theme will be elaborated on and illustrated with quotations from the interview data in the following sections, under four sub-themes: (1) agency in routine and daily prayer, (2) cognitive (re)framing through prayer, (3) prayer as a vehicle for active social interaction and support, and (4) prayer as work and transaction.

### Prayer as a space for active and agentic participation

Our analysis showed prayer to be a space for active and agentic participation for participants with dementia. Further in the text, we highlight what this entails under four sub-themes, which discuss active and agentic activities participants engaged in through prayer.

#### Agency in routine and daily prayers

Prayer formed a part of everyday life for participants in this study. The literature has shown that maintaining an everyday routine is important to living well with dementia (Andersen et al. 2004; Han et al. 2016). For participants in this study, prayer has been a major part of their everyday life – and remains a central aspect of their everyday life even with dementia. All participants in the study self-identified as religious, with 15 being Muslims – one of whom practised Islam together with a traditional Yoruba religion – and two being Christians, and they all spoke of prayer being a key routine activity they engaged in. Participants highlighted praying habitually five times a day. According to one Muslim participant:

‘I have faith in my act of worship. I pray five times a day. I don’t miss any of the prayers. I believe in God, and I believe that He will do whatever He wishes to do.’ (Baba Ade, M)

Participants’ agency was visible in how they negotiated details around prayer, such as when to pray, the choice to join communal prayers, and even the physical posture to assume when praying. For example, participants who lived with other physical disabilities that restricted their abilities to go to the mosque or church, perform the physical motions associated with Islamic prayers, or pray at the scheduled times, could decide for themselves how, when, and where to pray: ‘I usually do [*pray*]. Once they call for prayer, here that I am sitting, I will join in the prayer …’ (Mama Doyin, M). According to another participant:

‘… I usually go to the mosque before to pray… since this sickness [*limited mobility*] started, I have not been able to join them at the mosque to pray. So, I pray at home here. When I hear the call to prayer early in the morning, I will get up, do ablution, and pray here.’ (Baba Gbadebo, M, ATR)

Even participants whose religious traditions mandated the schedule for the daily prayer routine negotiated how and where they wanted to carry out the routine. Participants displayed both creativity and agency in these daily negotiations. For example, a Muslim participant who was blind, partially deaf, and had limited mobility stated:

‘I usually do the complete prayer [*i.e. pray five times a day*]. But I don’t do it at the originally scheduled time. If I pray in the morning and pray in the afternoon, I may not pray the other ones immediately with them. I will pray it all in the night when I am doing the final prayer.’ (Mama Niyi, M)

Participants with dementia also utilised prayer as an agentic tool – to express themselves and their wishes. While they majorly used prayer in this way when praying to God about themselves and their loved ones, they also used it as a way of asserting their agency during the interviews and conversations. They ‘interrupted’ with prayer as a way of asserting themselves, to clear up the air or say something they needed to say. For example, during an interview with a participant (Baba Seriki, M), his wife explained that he had been abandoned by his children because of his bad behaviour in the past. To defend himself against this perceived attack from his wife, Baba Seriki (M) cuts in with a prayer directed at the researcher: ‘Your secrets will not be uncovered. Your prayers will be answered. God will not put you in this condition. You will grow old …’ In another interview with a different participant, she had probably gotten tired of the discussion and wanted to let the first author know it was time to leave, so she started praying, ‘You will go and return safely … you will reap the fruit of your labour … thank you for coming to see me …’ (Mama Bosede, M). Sometimes, participants started to pray right in the middle of the interviews. Sometimes, it happened when they could not articulate an answer to a question being asked or simply did not want to answer. Other times, they added a prayer when there was silence – perhaps to fill the silence – or when we asked if they had anything else to say. For example, in response to a question about herself, a participant started praying for the research team: ‘Everything about you will be sweet! You will enjoy good things!’ (Mama Kike, M).

#### Cognitive (re)framing through prayer

Prayer was a routine that formed part of participants’ everyday lives, but its meaning and utilisation in participants’ lives were more than ordinary. For example, prayer allowed participants to build on their faith, strengthen their connections to God, and (re)frame their conditions. While being in the condition that most participants were in – poor, disabled, and living with dementia – may be a signal to an observer that participants were in unfortunate positions, they did not think of themselves this way. Many of the participants expressed gratitude to God for their lives and revelled in their connection to him, his response to their prayers, and their complete trust in his will. Take, for example, participants’ responses to common questions that were posed to them during each home visit or interview to inquire about their well-being and feelings of (un)happiness. Most participants with dementia answered such questions by thanking God for keeping them alive. One female participant said, ‘I thank God. I thank God for my life. Everything I am doing is okay. I appreciate God for my life. I am okay as I am now …’ (Mama Bosede, M). Another added:

‘I am always happy, and my heart is gladdened. Whenever I wake up, I am grateful to God that I woke up in peace; I thank him with my whole heart.’ (Mama Kike, M)

Many participants expressed contentment in their lives, which was rooted in their faith in God and their acceptance of his will. While it may be common in some faith traditions in Nigeria, such as the researcher’s Pentecostal Christianity, to see conditions such as illness and disability as a signal of wrongdoing against God or unanswered prayers, participants saw God’s answers to their prayer in the fact that they were alive and nothing ‘terribly bad has happened to’ them (Baba Ade, M). Another participant, when speaking of how she felt about her life, simply exclaimed, ‘Ahhh! God answers my prayers’ (Mama Jaiye, M). Dementia and other comorbidities were not seen as terrible situations that signalled a strain in their relationship with God or showed that their lives were bereft of answered prayers. This cognitive (re)framing shows how they viewed their relationships with God, what they interpreted as answered prayers, and how they coped with living with dementia. For example, one participant expressed, ‘God has given me life and peace … I am not bothered’ (Mama Niyi, M). Another added, ‘If I do not die … If I do not die, I believe the future will be better …’ (Mama Ibukun, C). Life and peace were, thus, framed as proof of God’s goodness in their lives and answers to their prayers. As long as they were alive, they knew that they were living a good life and that the future would be better. However, to some, even death was not a sign of being abandoned by God. These participants conceived of death as being called home to God. For example, when speaking of the death of his children, one of the participants stated:

‘God has called all my immediate children to his side … I don’t worry about the children that I lost, especially because they all had children, and I am happy each time I see them [*the grandchildren*].’ (Baba Ade, M)

They saw the dead person as reunited with God and the bereaved as standing with God on the other side of death. For example, when a participant was asked about her friends in the community, in trying to explain that all her friends were now dead, she expressed, ‘I am standing alone with God’ (Mama Kike, M). Another participant, speaking of being alone since her husband died, stated, ‘I don’t have another husband. I stand with God …’ (Mama Tani, M). This way of framing death and loss brought them to a place of acceptance and gratitude for what and who they still had with them.

Although many participants used prayer to negotiate with God for sustained well-being and improved conditions, ultimately, they accepted that God had the final say. And thus, framed difficult situations in their lives as part of God’s will. One participant, while speaking of her inability to walk and the possibility of walking again, stated:

‘It is God that made it that way. Even if I don’t like the situation, there is nothing I can do since God let it be like this … I cannot fully know the handwork of God. It may be possible.’ (Mama Yejide, M)

Another participant, while speaking of being abandoned by her children, stated, ‘That’s how their God created them to be … Ahhh! Their God created them that way’ (Mama Enitan, M). Participants acknowledged that they could do their parts of believing in God and making their supplications known to God, but it was all up to him to decide what to do, and they would be content with the decision. One participant expressed that although he believed in God and prayed five times a day without fail, he believed that God would do ‘whatever He wishes to do’ (Baba Ade, M). He went on to express:

‘It is only God that can say that [*decide the future*]. Whatever He brings, I will accept it. I cannot dictate for God … whatever God brings is what I will accept and appreciate.’ (Baba Ade, M)

#### Prayer as a vehicle for active social interaction and support

Social interaction, which has been highlighted in the literature as a protective factor for living with dementia (Han et al. 2016), was also found to be connected to prayer in this study. Prayer was more than just an individual and introspective activity for the participants; it was also an opportunity for social engagement and interaction. For participants, not only was praying daily an important routine but it was also essential for them to do this communally – going to the mosque or church and praying with others. In this sense, the practice of praying constituted everyday citizenship, as it confirmed a person’s identity as an active member of a religious community (see Hopkins & Blackwood 2011). Because of the impairment effects of dementia, Muslim participants did not always know when it was time to pray. However, this was not a problem for many because they could utilise resources around them to navigate this challenge of memory loss. For example, the *adhan* (the Muslim call to prayer) from the mosques around participants became an essential tool to navigate the challenges of memory problems and continue to participate in daily communal prayer: ‘When it is time for the prayer, they will shout and call everyone to prayer … their shouting makes me know that it is time to pray’ (Mama Kike, M). The call, thus, serves as a tool provided by the larger community, arguably unintentionally, to support people with dementia and encourage their continuous participation in communal practices.

Prayer and religious spaces connected participants to relationships and support outside their families. For example, those who went regularly to the mosque or church to pray found friendships there and enjoyed communal support from their faith community. This support or show of care sometimes came as visits to check in on them whenever they were absent from the mosque. According to one participant, ‘I always go to the Mosque, but any day I don’t go, the leaders of the Mosque will come to check and ask me what happened’ (Mama Kike, M). Another participant expressed getting visits from friends from the mosque whenever she was absent, ‘Ahhhhhhhh! Many, many of them! (laughs). They usually come from various places to see me … “ahh mama, you didn’t come to the mosque?”’ (Mama Jaiye, M). Prayer, thus, served as a vehicle through which participants found social support, a sense of belonging, and a sense of community, all of which are significant protective factors for dementia. Participants who could no longer go to the mosque or church to pray because of illnesses and disabilities were not left out of this show of care. One participant who was paraplegic spoke about receiving visits and monetary gifts from the Imams at the mosque:

‘They usually bring something. They will pray for me, asking God to make me stand up. One comes every seven days to pray for me and gives me money. Every day of Jimoh [*Friday*].’ (Mama Doyin, M)

Within these religious spaces, participants were not merely passive recipients of care and support, but active participants and providers of support themselves. Participants who went to the mosque and church to pray also had the opportunity to play active roles in these spaces. Some participants expressed that they participated in visiting and supporting other members of their faith community. According to a female participant:

‘There was one of my friends that stopped coming to the mosque; I went to see her at her house. I was asking her why she stopped coming to the Mosque.’ (Mama Kike, M)

Another participant explained how he played a key role in mobilising support for others in the mosque:

‘If there is any need to visit someone, I will gather people, and we will visit the person and pray for the person. If we want to go and visit the people that did not come to the mosque or that are sick, I will call all of them and lead them to the place …’ (Baba Ade, M)

The religious spaces also provided an opportunity for some participants to be part of key decision-making events and festivities outside of their families and homes:

‘It has been a while since we did such [*speaking of communal activities such as town hall meetings*] apart from the one in the Mosque; we did one at the Mosque three days ago and even two days ago. If there is anything to be done, we come together to discuss it and plan for how it will be done. Sometimes, they have a party there …’ (Baba Ade, M)

#### Prayer as work and transaction

Participants also used prayer to participate fully in their community and contribute to the lives of those around them. As stated earlier, many of the participants, in addition to dementia, had other disabilities and illnesses. This limited their abilities to work and participate in familial and communal lives in ways that they used to; thus, prayer now constituted much of their everyday activities and ‘responsibilities’. Some participants spoke about prayer as work – what they did now that they could no longer do their regular jobs. For example, one of the participants, who could no longer design local caps as he used to do in the past, expressed that leading prayers in the mosque and praying for people were his current work: ‘I am not doing anything apart from leading prayers for the people … that is the work I’m doing now’ (Baba Ade, M). Another participant who was paralysed expressed, ‘It is only prayer I have … it is only prayer that I have to offer. A prayerful mother is good! That is what I do’ (Mama Doyin, M)

Prayer was seen as a legitimate work by some participants, while others saw it as something they now had to do as they did not have much else going on. For example, one participant stated, ‘There is no other thing I am doing; there is nothing keeping me from praying’ (Mama Odunayo, M). For those who could not move around the community and could only sit or lie in their homes, prayer was used to stay ‘active’. One participant, when speaking of what she does to keep herself busy, stated, ‘Ahhh! It is prayer that I will be praying. It is prayer …’ (Mama Feyi, M).

Participants also used prayer in transactional ways – to repay kindness or give back to others. During the home visits, it was common for participants to pray for the researcher– and interpreter – before and after interviews. Before the researcher proceeded with the questions, participants loudly offered well wishes in the form of prayer. For example, while gearing up to start interviewing a participant, she started praying for the research team, ‘Nothing will happen to you …’ (Mama Jaiye, M). On another occasion, after chatting with a participant and giving her money in appreciation of her time, she started praying for the researcher and interpreter, ‘Toor! You will not suffer! You will go, and you will return; you will not meet any trouble’ (Mama Doyin, M). Another participant, after the interview, prayed thus:

‘God will bless you; your prayers will be answered … Ahhh. You will not be ashamed. God bless you. [*stops to ask if the researcher was married with kids, and after the researcher said no, continued*] God will give you a man of your choice … you will be blessed with children …’ (Mama Ronke, C)

This show of goodwill extended beyond the research relationship to encompass other people and relationships that mattered to them. Participants frequently prayed to God about the well-being of their family members and loved ones. According to one participant, ‘I pray for myself, my children, the senior wife, the senior wife’s children, and her husband’s family’ (Mama Enitan, M). Another participant, when probed about the content of her frequent prayers after she expressed how often she prayed, explained:

‘I thank God for making me be alive. I pray for my children and grandchildren; that they should prosper in all that they do … My children should go out in peace and return in good health.’ (Mama Kike, M)

Sometimes, the prayers extended beyond the living to even include people who were no longer alive:

‘I pray for so many people ooo. If I go to the mosque or am praying inside, I pray for my son, wife, children, grandchildren, and all the senior Imams that taught me the Quran who are still alive and even those who are dead. I pray for them. That the people that have died that God should have mercy on them.’ (Baba Ade, M)

Through the work of prayer, participants retained the roles of active agents and/or participants in their communities and found a useful tool with which to provide care and support to those around them. Sometimes, this brought them tangible rewards from those they prayed for. For example, some participants expressed that they sometimes received monetary or material gifts in exchange for praying for people in the community. According to one participant:

‘Many times, they pay me a little token for the prayer in appreciation … I use prayers to support outsiders [*non-family*] who come to me with challenges they need prayers to handle … and after doing the prayer for them, they give me a token.’ (Baba Ade, M)

Prayer had thus become, for some, an unintended relational transaction. A comment by another participant further illustrates this:

‘I usually pray for everyone, and they usually give me things. Do you understand? Everyone gives me something without begging them. Some of them would have gone a distance away, but they would still come back just to give me something. I don’t beg them ooooh!’ (Mama Doyin, M)

## Discussion

This article examines for the first time the role of religious faith and prayer in the everyday lives of people with dementia in Nigeria. Drawing on the accounts of a marginalised population whose lived experiences are underrepresented in both dementia and/or disability research and citizenship scholarship, the study has shown how religious practices, particularly faith and prayer, can become avenues for enacting and maintaining the everyday citizenship of people with dementia in Nigeria. Specifically, the major theme identified in participants’ accounts was that prayer served as a space for active and agentic participation. This theme was further elaborated upon through four subthemes: (1) agency in routine and daily prayer, (2) cognitive (re)framing through prayer, (3) prayer as a vehicle for active social interaction and support, and (4) prayer as work and transaction.

Participants described religious practices, such as faith in God, individual prayer, and communal prayer, as important to their acceptance of their situations, their feelings of hope in their everyday lives, and their connection and contributions to their community. Studies conducted elsewhere show that religion and spirituality are tools that bring acceptance, hope, and connection to people with dementia (Beuscher & Grando 2009; Katsuno 2003). Beuscher and Grando’s (2009) study, which primarily focused on Christians, reported that although most participants’ faith or beliefs were not affected by their cognitive impairment, it affected their participation in religious activities such as praying and being involved in the church. This was, however, not the case in this study. Participants, a majority of whom identified as Muslims, reported continued involvement in religious activities – they prayed daily and went to religious spaces almost regularly. For Muslim participants, this was facilitated by the proximity of mosques to people’s homes, the call to prayer (adhan), which reminded them when it was time to pray, and the support they got from friends and leaders who regularly followed up on them.

Faith and prayer have been shown to play a positive role in the experiences of people with dementia in this Nigerian community. For participants, religious practices, such as prayer, had become more than a spiritual meaning-making tool for people with dementia and were transformed into a space for active ageing, asserting agency, participating in everyday and communal life, dealing with loss and other challenges, and supporting and being supported by others. This invariably has implications for dementia theorisation, care, and research. Taking a departure from the findings from this study, policymakers, scholars, and activists in Nigeria and Africa can re-imagine a future for dementia care where not only both indigenous African and non-African but contextualised religious practices can be also incorporated into care and structures of everyday living. For example, the way that participants with dementia in this study asserted their wishes and needs through praying about them can provide insight for dementia care workers on the different ways by which people with dementia may communicate their needs. Research studies that encourage participants with dementia to express themselves through praying out loud to God rather than talking directly to an interviewer may uncover different needs and wishes of people with dementia.

Our analysis shows the centrality of relationality in the everyday experiences of people with dementia, highlighting the embodied, agentic, and participatory nature of relationality in and through religious spaces and practices. Through relationships formed and maintained by prayer, going to the mosque or church, and giving and receiving supportive visits, people with dementia actively participate as full members of their communities. Relationality, within the context of dementia research, has been widely explored from scholars such as Adams and Gardiner (2005) and Ryan et al. (2008), who developed the paradigm of relationship-centred care, to scholars such as Bartlett and Connor (2010), Kontos et al. (2016), Kontos, Miller and Kontos (2017), who have expanded the work on citizenship in dementia studies. However, while the centrality of relationships in the dementia experience has been explored and established by many dementia scholars, these works have been mainly limited to human interconnectedness. The literature has not emphasised connections to and through religious practices, spaces, and deities. A major contribution of this study lies in this limitation in the literature. The findings show how relationships with a supernatural being, maintained through routine prayer, can not only impact how people with dementia frame themselves and their situations but also impact the relationships that they have with others in their community. Policymakers, researchers, and dementia care workers can use prayer and religious spaces as tools for exploring the experiences and relationships of people with dementia.

Scholarly understandings of dementia and approaches to dementia care in Nigeria are significantly contextualised within a biomedical and secular framework. Those working within a biomedical framing tend to understand dementia in terms of an illness or health condition that needs to be monitored, diagnosed, understood, and managed pharmaceutically and/or therapeutically (see, e.g., Amoo et al. 2011; Ochayi & Thacher 2006; Perkins et al. 2002). There is, however, a wealth of resources to unravel and utilise in dementia care when we consider experiences of dementia contained within the everyday communal and religious space. For example, rituals of daily personal and communal prayers and visitations, as seen in this study, provide persons with dementia supportive structures and activities to look forward to and participate in as full community members. Religious activities even become avenues for work and inclusion in communal and everyday life for those unable to work anymore. This has an implication for scholars and professionals who aim to improve the well-being of people with dementia. The temporal practices attached to Islam, for example, such as the calls to prayer (adhan), which was shown to help participants in this study tell time and join in communal prayers, can also be a resource to note when designing or advocating for dementia care in Nigeria and other contexts where Muslim people with dementia live.

The spiritualisation of dementia in Africa is often regarded negatively, such as instances where people with dementia are considered witches (Adebiyi et al. 2016; Brooke & Ojo 2020; Khonje et al. 2015; Mkhonto & Hanssen 2018; Mushi et al. 2014; Ndamba-Bandzouzi et al. 2014). However, as the results of this study have shown, religious beliefs and practices do not always necessarily affect the lives of people with dementia negatively. As other researchers have shown, faith healers may not perceive and understand dementia as an illness, but they have their place in caring for people with dementia (Hindley et al. 2017). Participants in our study did not conceptualise dementia as an illness or impairment in the same way they did other multimorbidities they lived with; however, we do not interpret this as an error that needs to be corrected through education or awareness. An alternative – or rather, community-based – approach to understanding dementia can be a rich resource to explore to advance theoretical understandings of dementia and practice-based approaches to dementia care.

## Conclusion

This article contributes to advancing the understanding of the socially orientated everyday experience of dementia in Nigeria and Africa, drawing on narratives of everyday religious practices from people with dementia themselves. It contributes to a small body of literature on the social, everyday experiences of living with dementia in Africa and stands as the first of such a study in Nigeria. However, it does not represent the experiences of different groups of people with dementia in Nigeria. Further research is needed to highlight the experiences of people with dementia from other tribes/ethnic groups, religions, geographical locations, and socio-economic backgrounds in Nigeria.
